# Hypoxia-Induced Reactive Oxygen Species Cause Chromosomal Abnormalities in Endothelial Cells in the Tumor Microenvironment

**DOI:** 10.1371/journal.pone.0080349

**Published:** 2013-11-15

**Authors:** Miyako Kondoh, Noritaka Ohga, Kosuke Akiyama, Yasuhiro Hida, Nako Maishi, Alam Mohammad Towfik, Nobuo Inoue, Masanobu Shindoh, Kyoko Hida

**Affiliations:** 1 Department of Vascular Biology, Hokkaido University Graduate School of Dental Medicine, Sapporo, Japan; 2 Department of Oral Pathology and Biology, Hokkaido University Graduate School of Dental Medicine, Sapporo, Japan; 3 Department of Gerodontology, Hokkaido University Graduate School of Dental Medicine, Sapporo, Japan; 4 Department of CardioVascular and Thoracic Surgery, Hokkaido University Graduate School of Medicine, Sapporo, Japan; Osaka University Graduate School of Medicine, Japan

## Abstract

There is much evidence that hypoxia in the tumor microenvironment enhances tumor progression. In an earlier study, we reported abnormal phenotypes of tumor-associated endothelial cells such as those resistant to chemotherapy and chromosomal instability. Here we investigated the role of hypoxia in the acquisition of chromosomal abnormalities in endothelial cells. Tumor-associated endothelial cells isolated from human tumor xenografts showed chromosomal abnormalities, >30% of which were aneuploidy. Aneuploidy of the tumor-associated endothelial cells was also shown by simultaneous in-situ hybridization for chromosome 17 and by immunohistochemistry with anti-CD31 antibody for endothelial staining. The aneuploid cells were surrounded by a pimonidazole-positive area, indicating hypoxia. Human microvascular endothelial cells expressed hypoxia-inducible factor 1 and vascular endothelial growth factor A in response to either hypoxia or hypoxia-reoxygenation, and in these conditions, they acquired aneuploidy in 7 days. Induction of aneuploidy was inhibited by either inhibition of vascular endothelial growth factor signaling with vascular endothelial growth factor receptor 2 inhibitor or by inhibition of reactive oxygen species by N-acetyl-L-cysteine. These results indicate that hypoxia induces chromosomal abnormalities in endothelial cells through the induction of reactive oxygen species and excess signaling of vascular endothelial growth factor in the tumor microenvironment.

## Introduction

Angiogenesis is a physiological process involving growth of new blood vessels, and it is necessary for tumor progression and metastasis. Tumor blood vessels provide nutrition and oxygen and eliminate waste from tumor tissue, resulting in tumor progression [1]. In the tumor microenvironment, tumors also exhibit nutrient deprivation, an acidic extracellular pH, high interstitial pressure, and excess pro-angiogenic activity such as vascular endothelial growth factor (VEGF) activity [2]. Hypoxia regulates tumor angiogenesis by modulating a large number and variety of pro- and anti-angiogenic factors [3,4]. Regulation of genes that encode proteins involved in angiogenesis occurs through the activation of hypoxia-inducible factor (HIF). HIF, a heterodimeric complex comprising α and β subunits, is a specific DNA-binding protein that affects the transcription of proangiogenic genes [5].

One of the most versatile angiogenic factors stimulated by hypoxia is VEGF [4,6], which is induced and regulated in a strictly dose-dependent manner by HIF-1 [7–9]. Therefore, tumor endothelial cells (TECs) are exposed to an extracellular environment that is markedly different from that of endothelial cells (ECs) resident in healthy normal tissue (normal endothelial cells; NECs). ECs within the tumor microenvironment are sometimes exposed to hypoxia [2,10]. However, there are few detailed reports on the response of TECs to hypoxia. Traditionally, TECs were believed to be genetically stable, but recent studies suggest that TECs are different from NECs. For example, TECs are more angiogenic, and the expression of several genes such as COX-2 [11], VEGF [12,13], and VEGF receptor 2 (VEGFR-2) is upregulated [14]. Furthermore, we found that TECs are cytogenetically abnormal [15-17]. However, the mechanisms of TEC aneuploidy are not yet understood. Unraveling this mystery would provide a significant breakthrough in understanding how ECs become genetically abnormal in the tumor microenvironment.

The hypoxic condition in tumor tissue is known to induce genetic alterations through the induction of genetic instability [18]. Moreover, the oxygen concentration within a hypoxic region is highly variable. Because tumor vasculature is highly immature and unstable, red blood cells flux to the hypoxic regions, resulting in reperfusion or reoxygenation [19]. Hypoxia and reoxygenation induce oxidative stress in cells [20]. Reactive oxygen species (ROS) are often considered as harmful metabolic products and have traditionally been implicated in the pathogenesis of cardiovascular diseases and cancer. ROS can cause damage to cellular macromolecules and lead to increased genetic instability [21,22]. Cell stress induced by the microenvironment, particularly hypoxia and reoxygenation, may cause these genetic changes [23,24]. Therefore, we hypothesized that hypoxia in the tumor microenvironment can induce chromosomal abnormalities in ECs.

In this study we investigated the involvement of hypoxia-induced ROS in the generation of TEC abnormalities.

## Materials and Methods

### Cell lines and culture conditions

A375-SM cells (supermetastatic human malignant melanoma cells) were a gift from Dr Isaiah J. Fidler (MD Anderson Cancer Center, Houston, TX) [25]. Human microvascular ECs (HMVECs) were purchased from Lonza (Tokyo, Japan). HSC-3 cells were purchased from Riken. A375-SM cells were cultured in minimum essential medium (Gibco, Grand Island, NY) supplemented with 10% heat-inactivated fetal bovine serum (FBS), and HSC-3 cells were cultured in Dulbecco’s modified Eagle medium (Gibco) supplemented with 10% FBS. HMVECs were cultured in EC growth medium for microvascular cells (EGM-2MV; Lonza, Basel, Switzerland) in a humidified atmosphere of 5% CO_2_ and 95% air at 37°C. Hypoxic culture conditions were achieved using a multigas incubator containing a mixture of 94% N_2_, 5% CO_2_, and 1% O_2_ (ASTEC, Fukuoka, Japan). HMVECs were subjected to 24 h of hypoxia (1% O_2_), 24 h of normoxia (20% O_2_), and 24 h of hypoxia followed by 24 h of normoxia (hypoxia-reoxygenation) for 7 days.

### Antibodies

The following antibodies were used: FITC–anti-mouse CD31, anti-mouse (BD Pharmingen, San Diego, CA), anti-HIF-1α (Cell Signaling Technology, Danvers, MA), anti-human carbonic anhydrase ix (CA ix; R&D Systems, Minneapolis, MN), anti-HIF-1α (Cayman), and monoclonal anti-β-actin (Cell Signaling), anti-Ki67 (abcam, Cambridge, MA)

### Isolation of TECs and NECs

All procedures for animal experiments were performed following the regulation on animal experimentation of Hokkaido University. This study was approved by the Animal Care and Use Committee of Hokkaido University (approval ID: 08-0296). Mouse TECs (mTECs) and NECs (mNECs) were isolated as previously described with some modifications [11,13,16,17,19,26-30]. In brief, mTECs were isolated from human tumor xenografts (A375-SM and HSC-3) in nude mice. mNECs were isolated from normal mouse dermis as control. After tissue digestion, dissociated cells were incubated with rat anti-mouse CD31 (BD Pharmingen). CD31-positive cells were sorted using a magnetic activated cell sorting system (Miltenyi Biotec, Tokyo, Japan) according to the manufacturer’s instructions. Diphtheria toxin (DT; 500 ng/milliliter; Calbiochem, San Diego, CA) was added to mTEC subcultures to kill any human tumor cells (DT was also added to mNEC subcultures for technical consistency).

### Cell proliferation assay

For each condition, HMVECs (5 × 10^4^ cells) per well were seeded in a 6-well plate to analyze cell proliferation. ECs were cultured under normoxia, hypoxia, and hypoxia-reoxygenation for 1 week. The number of cells was counted 1 week after seeding. Data are presented as the average of three well counts under the respective conditions.

To analyze cell proliferation, mouse TECs and NECs were seeded onto a plate (2 × 10^4^ cells). Cell number was counted every day for 4 days. Each experiment was performed in triplicate, with similar results. 

### Western blotting

HMVECs were harvested under normoxic and hypoxic conditions (1% O_2_) for 24 h. After 24 h, HMVECs were lysed as described previously. Total protein was measured using a BCA protein assay kit (Pierce, Rockford, IL, USA). Western blot analysis was performed using antibodies specific to HIF-1α, β-actin, and horseradish peroxidase (HRP)-conjugated secondary antibodies, as described previously [13,29]. 

### Real-time PCR analysis

Total RNAs were reverse transcribed using ReverTraAce (Toyobo, Osaka, Japan) as previously described. For relative quantification of target mRNA, SsoFast™ EvaGreen^®^ Supermix quantitative PCR detection (CFX96 Real-Time PCR detection System; Bio-Rad, Hercules, CA) was used for mouse ECs and SYBR^®^ Green Real-Time PCR Master Mix-Plus (Bio-Rad) was used for human ECs (in triplicate) according to the manufacturers’ protocols. Quantitative PCR was performed at 95°C for 3 min, followed by 45 cycles at 95°C for 10 s and 60°C for 30 s. Data were analyzed with CFX Manager software (Bio-Rad). Target gene expression was quantified by determining the threshold cycle [31]. Four PCR reactions were used in each experiment, and each experiment was performed in triplicate. The primers used are shown in Figure S1.

### Cell cycle analysis

HMVECs were cultured under normoxic, hypoxic, and hypoxia-reoxygenation conditions and prepared for analysis following the instructions of the Cycletest Plus DNA Reagent Kit (BD, San Jose, CA). After staining of cells with propidium iodide solution for 30 min, data for 100,000 events was obtained using Aria-2 (BD). Distribution of cells in the different phases of the cell cycle was analyzed from the DNA histograms using FACS and FlowJo software (Ashland, TreeStar).

### Detection of ROS production using flow cytometry

Intracellular ROS level was detected using the Total ROS/Superoxide detection kit (Enzo Life Science, Plymouth Meeting, PA). HMVECs were cultured under conditions of normoxia, hypoxia, or hypoxia-reoxygenation for 1 week. Cells were harvested and stained with an oxidative stress detection reagent and analyzed by flow cytometry (Aria-2; BD). The experiments were performed in triplicate with similar results. 

### Immunocytochemistry

Cells were cultured on coverslips under the different conditions in a 6-well plate, fixed in 4% paraformaldehyde for 10 min, washed with PBS for 5 min, and then permeabilized with Triton X-100 (Sigma-Aldrich). The cells were incubated with anti-HIF-1α overnight at 4°C, followed by incubation with secondary antibody. Nuclei were stained with 4’, 6-diamino-2-phenylindole (DAPI; Roche Diagnostics, Penzberg, Germany). Stained samples were visualized using a confocal microscope (FluoView FV1000; Olympus).

### Fluorescence in-situ hybridization (FISH)

Mouse ECs were directly isolated from tumor or normal tissues (high purity, 95%) and analyzed by FISH. After immunostaining with FITC-conjugated CD31 (noncultured ECs) or staining with FITC-conjugated BS1-B4 (cultured ECs), slides were fixed for 45 min using Histochoice (AMRESCO, Solon, OH) as previously described [18,19]. FISH was performed using a Cy3-mouse chromosome-17 locus-specific A1 probe (RP23-146B6; Chromosome Science, Sapporo, Japan). All samples were counterstained with DAPI. Hybridization signals were observed and analyzed using an Olympus IX71 fluorescence microscope. For HMVECs exposed to hypoxic conditions, FISH was performed using a Cy3-mouse chromosome-7 locus-specific probe (clone name, RP11-81B20, RP11-815K24: 7p11) and a chromosome-8 locus-specific probe (clone name, RP11-366N9, RP11-947H23, RP11-1077A8: 8p11; Chromosome Science). Chromosomes were counted in at least 100 nuclei for each sample. The probe was tested before analysis using normal peripheral blood mononuclear cells. After confiming that aneuploidy was smaller than 5% (in normal range), the probe was used for further analysis. Aneuploid cells were counted three times in each sample. Cells with a single signal for each probe were not included in the analysis because it was difficult to judge whether the single signal was due to monosomy or incomplete hybridization.

### Assessment of tissue hypoxia

Hypoxic areas in high-metastatic tumor cryosections and muscle tissue (normal tissue) were detected using the Hypoxyprobe-1 TM Kit (HPI, Burlington, MA). In brief, 60 mg/kg pimonidazole was injected intraperitoneally 30 min before the animal was killed, and samples were stained with Hypoxyprobe (FITC-conjugated anti-pimonidazole mouse monoclonal antibody). Resected tumor tissue was immediately immersed in liquid nitrogen. Frozen sections (thickness, 7 μm) were double-stained using Alexa Fluor 594-anti-CD31. To analyze the hypoxic area in high-metastatic tumor and muscle tissue, stained samples were visualized under a FluoView FV1000 confocal microscope (Olympus). FISH was also performed in tumor sections [17]. All IHC samples were counterstained with DAPI and visualized under an Olympus IX71 fluorescence microscope and an Olympus FluoView FV1000 confocal microscope in each experiment.

### Karyotype analysis

Karyotype analysis of HMVECs subjected to conditions of normoxia, hypoxia, and hypoxia-reoxygenation was performed by Q banding, as previously described [32]. Five karyotypes of HMVECs were analyzed for each condition, and 20 metaphase spreads were counted.

### Statistical analysis

Differences between groups were evaluated using Student’s t-test. A p-value of <0.05 was considered significant, and p < 0.01 was considered statistically significant.

## Results

### TEC characterization and relationship with hypoxia

**Figure 1:**
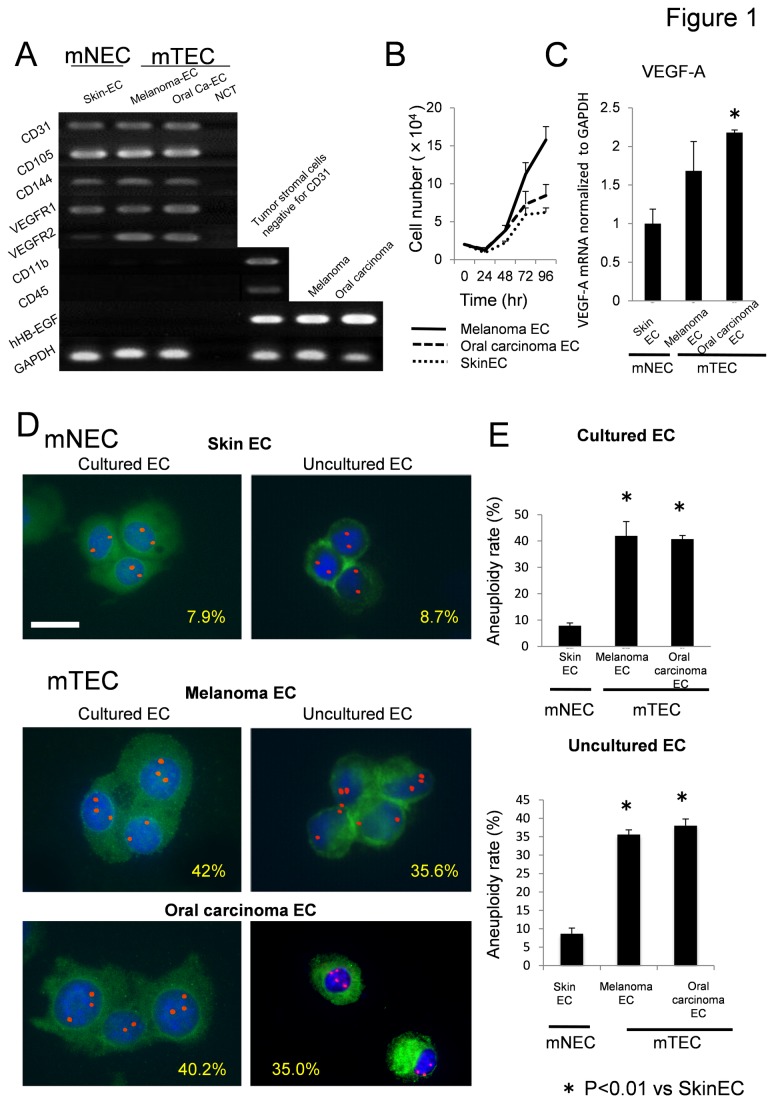
Tumor endothelial cell (TEC) characteristics. (A) Cultured NECs and TECs are positive for CD31, CD105, CD144, VEGFR1, and VEGFR2 and negative for CD11b and CD45. Human HB-EGF expression is shown in CD31 negative cells and human tumor cells (Melanoma and Oral carcinoma: Oral-Ca) using a human primer, but not in TECs. Abbreviation: NCT = Negative Control Template. (B) TECs showed a higher proliferation rate compared with NECs. (C) Expression levels of the angiogenesis-related gene VEGF in ECs by real-time PCR. Expression levels of VEGF were higher in TECs than in NECs. (D) Cultured ECs were stained with FITC-conjugated BS1-B4 lectin, and FISH was performed using a Cy3-mouse chromosome-17 locus-specific BAC probe. Red, chromosome 17; green, BS1-B4. FISH revealed aneuploidy in 42% melanoma ECs, 40.2% oral carcinoma ECs, and 7.9% NECs. Freshly isolated and noncultured TECs were cytospun onto a glass slide and immunostained for CD31 (green) and subjected to FISH. Aneuploidy was observed in 35.6% melanoma ECs, 35.0% oral carcinoma ECs, and 8.7% NECs. Scale bar, 10 μm. Experiments were performed in triplicate. *p < 0.01.

TECs were isolated from human tumor xenografts in nude mice (oral carcinoma, melanoma), as described previously with some modifications [11,13,15-17,29,33,34]. NECs were isolated from the dermis (skin ECs) and used as controls. RT-PCR revealed that NECs and TECs were positive for EC marker CD31, CD105, CD144, VEGFR-1, and VEGFR-2 and negative for the monocyte marker CD11b and the hematopoietic marker CD45, indicating that these ECs possessed EC characteristics during culture. Because we used DT, which binds hHB-EGF and can kill human cells, cultured TECs were not contaminated with human tumor cells [15, 16, 28, 29, 35] (Figure 1A). TECs showed a higher proliferation rate compared with NECs (Figure 1B). We compared the expression levels of the angiogenesis-related gene VEGF in ECs by real-time PCR. Expression levels of these genes were higher in TECs than in NECs (Figure 1C).

We previously reported that murine TECs were karyotypically aneuploid, whereas NECs cultured under the same conditions were diploid. After ECs were cultured, FISH revealed that TECs showed a significantly higher rate of aneuploidy (42.0% of melanoma ECs and 40.2% of oral carcinoma ECs) compared with NECs (8%). Freshly isolated and noncultured TECs were cytospun and immunostained with CD31 (green) and subjected to FISH. FISH using a mouse chromosome-17 probe revealed that noncultured TECs showed a significantly higher rate of aneuploidy (35.6% of melanoma ECs and 35.0% of oral carcinoma ECs) compared with NECs (8.7%; Figure 1D,E). We previously reported that TEC aneuploidy was increased in culture [16,17]. These results confirmed previous findings of chromosomal abnormalities in TECs [15-17].

### Hypoxia induces VEGF expression in HMVECs

**Figure 2:**
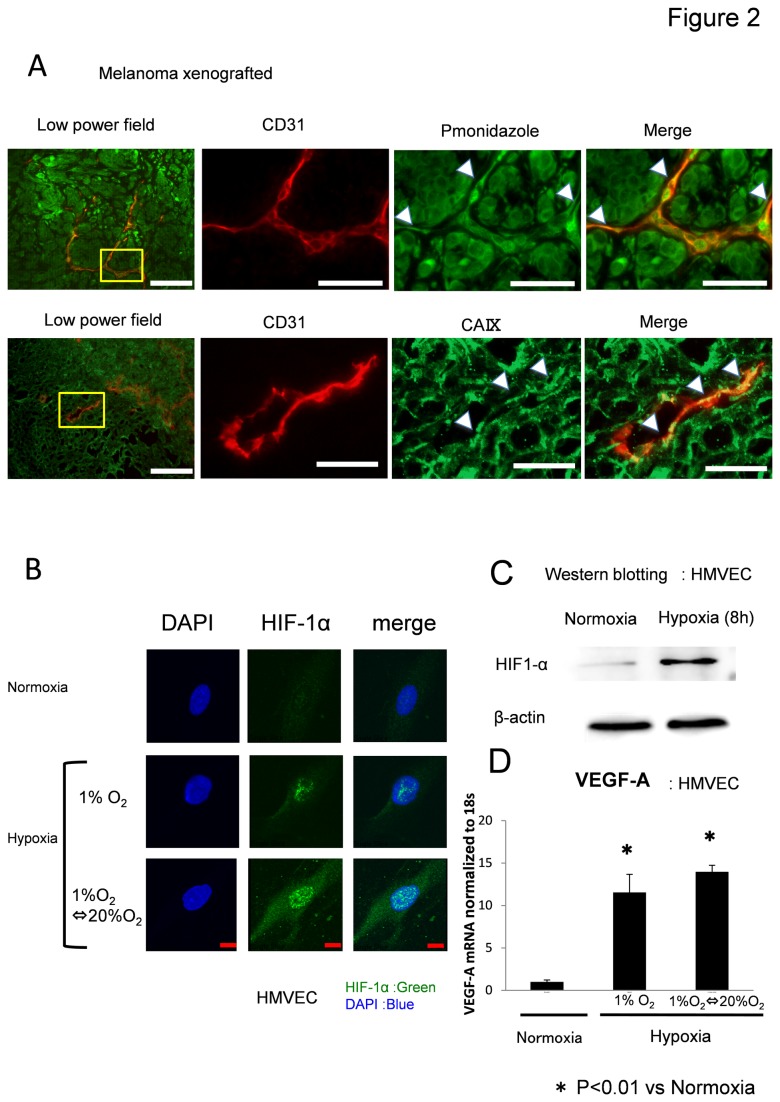
Hypoxia induces HIF-1α and VEGF-A expression in ECs. (A) The hypoxic area in human tumor xenografts in nude mice was analyzed using the hypoxia marker pimonidazole and CA IX. Tumor tissues were double-stained with anti-CD31 (red) and anti-pimonidazole antibodies or anti-CA IX (green) to visualize hypoxic areas. Pimonidazole staining revealed that tumor vessels were exposed to hypoxia to some extent. Scale bar, 100 μm. (B) HMVECs were cultured and treated for 8 h under normoxia or hypoxia. HIF-1α protein was upregulated 8 h after hypoxia, as revealed by western blotting. Densitometry analysis revealed that HIF-1α was induced by hypoxia. (C) HMVECs were cultured and treated for 8 h under normoxia or hypoxia. HIF-1α protein was upregulated 8 h after hypoxia, as revealed by western blotting. Densitometry analysis revealed that HIF-1α was induced by hypoxia. The experiment was repeated three times. Representative data is shown. (D) mRNA levels of VEGF-A were significantly increased by hypoxia in HMVECs. Experiments were performed in triplicate. *p < 0.01.

Hypoxia stimulates angiogenesis-related genes, which regulate diverse cellular processes, including tumor angiogenesis. Subsequently, the hypoxic area in tumors was examined using the hypoxia marker pimonidazole and CA IX. Tumor tissues were double-stained with anti-CD31 and anti-pimonidazole antibodies or anti-CA IX antibodies to visualize hypoxic areas. Pimonidazole or CA IX staining revealed that a large area of tumor was exposed to hypoxia (mouse *n* = 3; Figure 2A). Furthermore, even tumor blood vessels were exposed to hypoxia. Next, the effects of hypoxia on TECs were investigated. Exposure of cultured HMVECs to hypoxia caused expression of HIF-1α protein within 24 h. Induction of HIF-1α protein expression was confirmed by immunofluorescent staining of cultured ECs under conditions of normoxia or hypoxia (Figure 2B). Expression of HIF-1α was significantly upregulated after 8 h of hypoxia (Figure 2C). The effects of hypoxia on the expression of VEGF-A by cultured HMVECs were determined by RT-PCR. The mRNA levels of VEGF-A significantly increased by hypoxia (Figure 2D). These results suggest that the proangiogenic phenotype of TECs may be caused by HIF-1α and VEGF upregulation, which may result from hypoxia in the surrounding blood vessels in an in-vivo microenvironment. 

### Hypoxia induces aneuploidy in HMVECs

We previously reported that murine TECs were karyotypically aneuploid, whereas NECs cultured under the same conditions were diploid [15-17]. Aneuploidy was demonstrated in human renal carcinoma-derived TECs [17].

TECs were shown to have more angiogenic properties compared with NECs [28,29]. TECs showed a higher proliferation rate and greater migration toward VEGF. Expression levels of the angiogenesis-related genes VEGF, VEGFR-1, and VEGFR-2 were upregulated in TECs compared with those in NECs [15,28]. Intratumoral hypoxia in the tumor microenvironment stimulates VEGF signaling, which regulates diverse cellular processes, including tumor angiogenesis [7].

**Figure 3:**
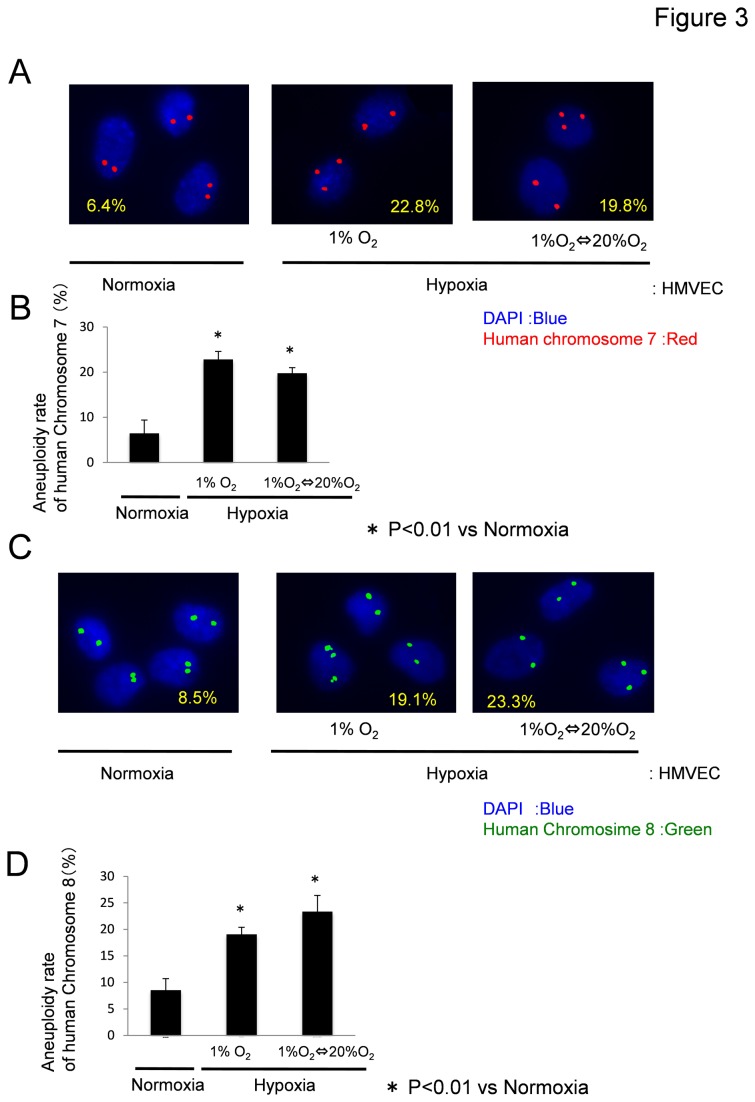
Hypoxia induces aneuploidy in HMVECs. (A, B) After HMVECs were cultured in each condition for 7 days, FISH analysis using a chromosome-7 probe revealed that approximately 22.8% ECs in the hypoxic condition (1% O2) and 19.8% ECs in the hypoxia-reoxygenation condition were aneuploid, whereas 6.4% ECs in the normoxic condition were aneuploid. (C, D) FISH analysis using a chromosome-8 probe revealed that approximately 19.1% ECs in the hypoxic condition and 23.3% of ECs in the hypoxia-reoxygenation condition were aneuploid, whereas 8.5% ECs in the normoxic condition were aneuploid.

In addition, elevated VEGF signaling leads to centrosome overduplication, and blockade of relevant pathway components rescues the centrosome duplication defect [37]. We hypothesized that hypoxia and elevated VEGF signaling may induce chromosome missegregation, causing aneuploidy in ECs. Cytospin ECs were subjected to FISH using a human chromosome-7 probe and a chromosome-8 probe. FISH analysis with the chromosome-7 probe revealed that approximately 22.8% ECs in the hypoxic condition and 19.8% ECs in the hypoxia-reoxygenation condition were aneuploid. On the other hand, 6.4% ECs in the normoxic condition were aneuploid when the chromosome-7 probe was used (Figure 3A,B). In addition, FISH analysis using the chromosome-8 probe revealed that approximately 19.1% ECs in the hypoxic condition and 23.3% ECs in the hypoxia-reoxygenation condition were aneuploid, whereas only 8.5% ECs in the normoxic condition were aneuploid (Figure 3C,D). Since HMVECs showed aneuploidy (5-9%) even just after purchased, it was considered that our FISH results of normoxia HMVECs were caused by cell culture artifact. 

### HMVECs acquire an abnormal karyotype under hypoxic conditions

**Figure 4:**
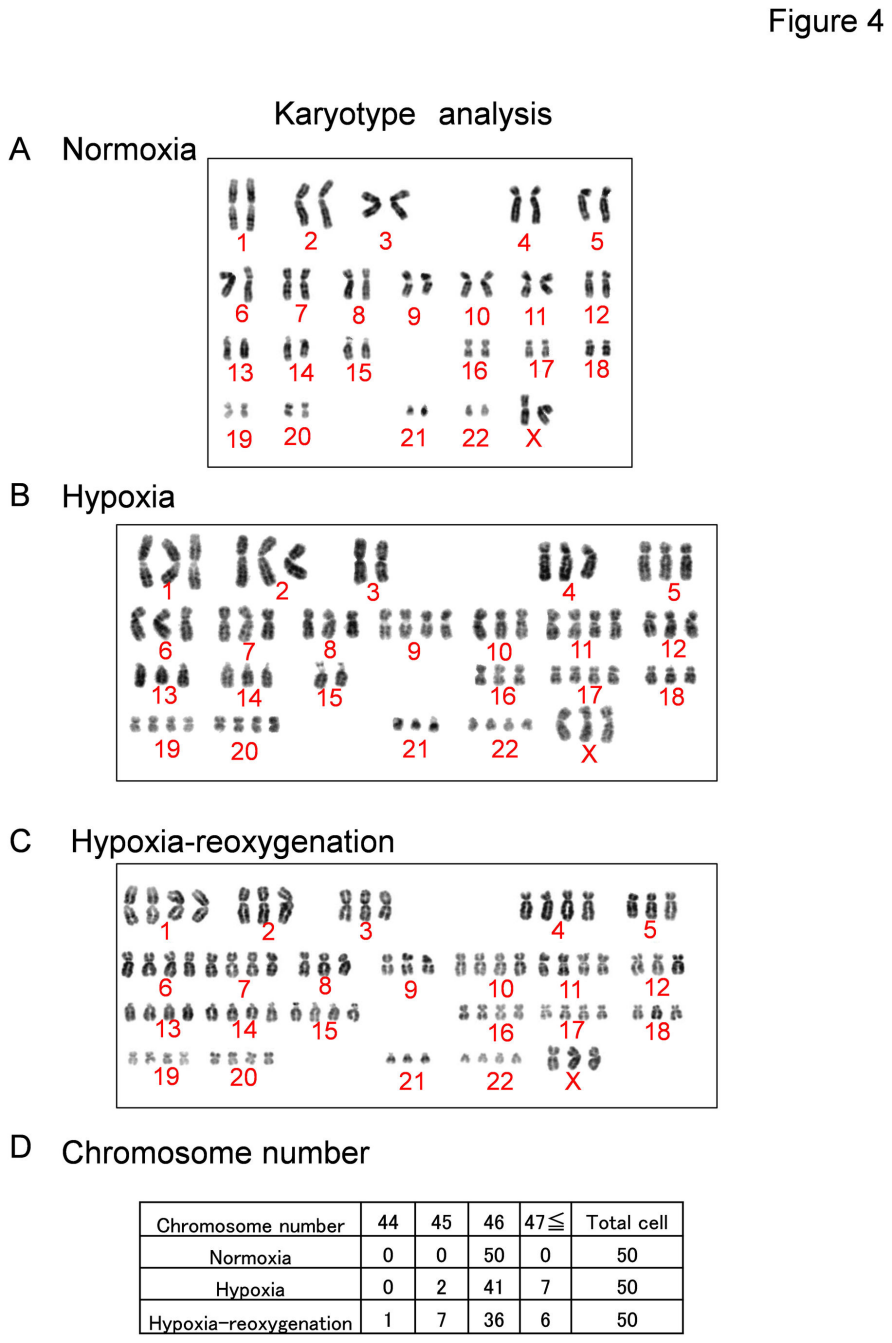
Karyotypes of HMVECs exposed to hypoxia. Karyotypes of HMVECs after exposure to normoxia, hypoxia (1% O2), and hypoxia-reoxygenation were analyzed by Q band analysis. A karyotype of one cell in each condition is shown. (A) HMVECs in normoxia were essentially diploid. (B, C) HMVECs exposed to hypoxia and hypoxia-reoxygenation had complex abnormal karyotypes and were aneuploid. (D) The chromosome number of each condition was counted and shown.

Karyotypes of HMVECs were analyzed by Q banding. Representative images are shown in Figure 4. HMVECs exposed to hypoxia had more complex and abnormal karyotypes compared with HMVECs exposed to normoxia. HMVECs under hypoxia demonstrated several missing chromosomes, markers of unknown origin, and an abnormal number of chromosomes (Figure 4A–C). Hypoxia caused chromosomal abnormalities of NECs (hypoxia, 18%; hypoxia-reoxygenation, 28%; Figure 4D). 

### Assessment of tumor hypoxia in vivo and FISH in tumor sections

**Figure 5:**
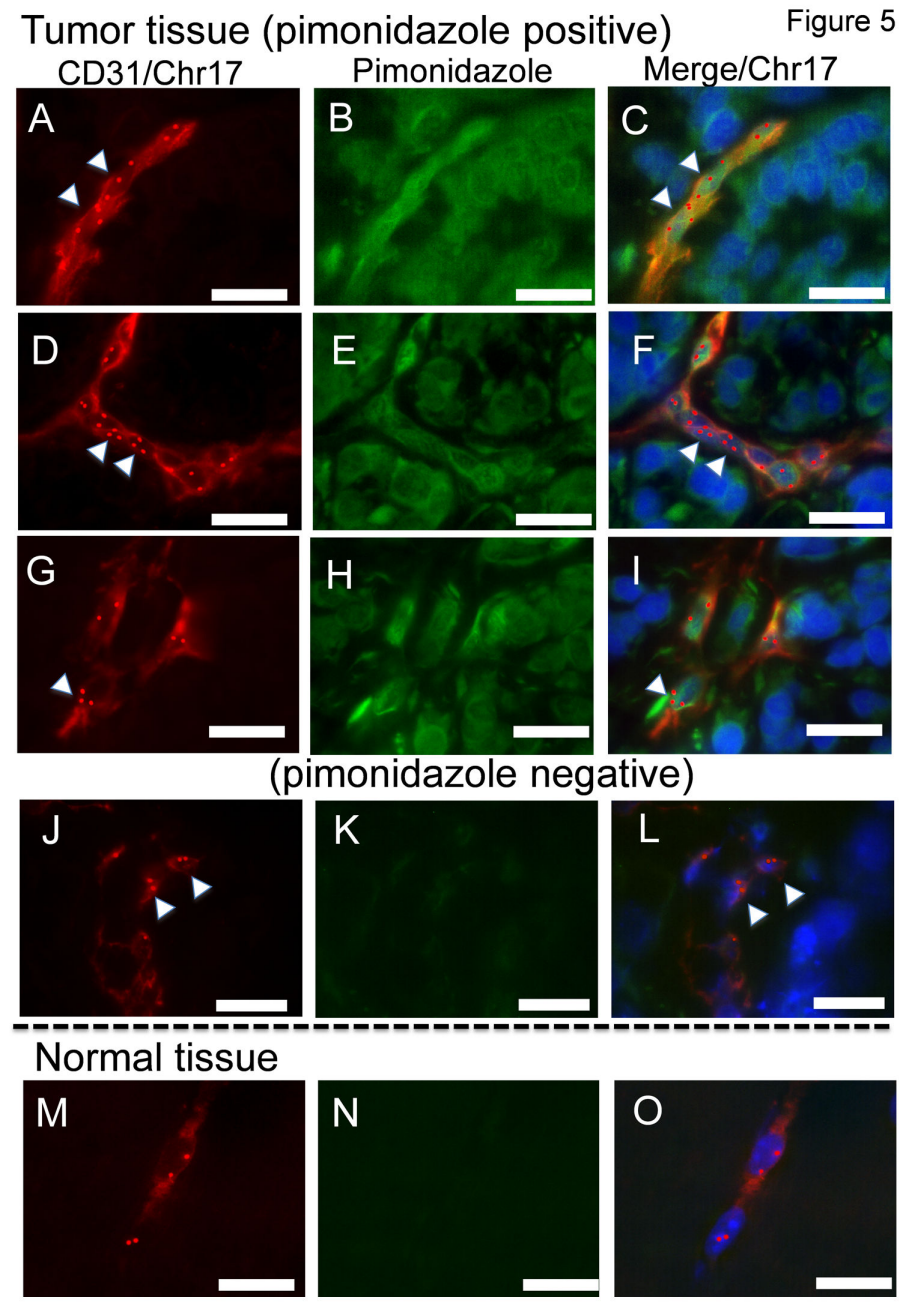
Assessment of tumor tissue hypoxia and analysis of ploidy in TECs by FISH Hypoxic areas in supermetastatic tumor cryosections were detected using hypoxyprobe. To analyze the hypoxic area in TECs in in-vivo tumors, frozen sections of tumors were immunostained with anti-CD31 (red) and anti-pimonidazole (green) followed by FISH using a spectrum red-conjugated mouse chromosome-17 locus-specific probe (red spot). Representative CD31-positive ECs in tumor tissue. (A, D, G) The blood vessels in tumor tissue were pimonidazole-positive. (B, E, H) Nuclei are stained with DAPI (blue). Aneuploidy was also observed in ECs of tumor blood vessels exposed to hypoxia. (C, F, I) Pimonidazole-nagative ECs in tumor tissue did not exhibit aneuploidy. (J, K, L) ECs in normal tissue did not exhibit aneuploidy. (M, N, O) Scale bar, 20 μm.

The relationship between hypoxia and aneuploidy in TECs in tumors *in vivo* was analyzed by immunostaining and FISH. The hypoxic area in tumor tissue (human supermetastatic tumor xenografted model) was analyzed using the hypoxia marker pimonidazole. Immunostaining was performed to identify microvascular ECs with anti-CD31 antibody, followed by FISH using a mouse chromosome-17 probe. Figure 5A, D, G, J, M shows representative CD31-positive ECs in tumor tissue. Tumor tissues were double-stained with anti-CD31 and anti-pimonidazole antibodies to visualize ECs exposed to hypoxia. Most of the blood vessels in tumor tissue (supermetastatic tumors) were pimonidazole-positive (Figure 5B, E, H). Aneuploidy was also observed in ECs of tumor blood vessels exposed to hypoxia (Figure 5C, F, I). In contrast, ECs in pimonidazole-negative tumor blood vessels were diploid (Figure 5J, K, L). Furthermore, in normal tissue (muscle tissue) did not exhibit aneuploidy (Figure 5M, N, O) . This suggests that the tumor blood vessels are exposed to hypoxia in some area and that TECs may have chromosomal abnormalities (mouse, *n* = 3).

### Cell-cycle analysis of NECs under the hypoxic condition

**Figure 6:**
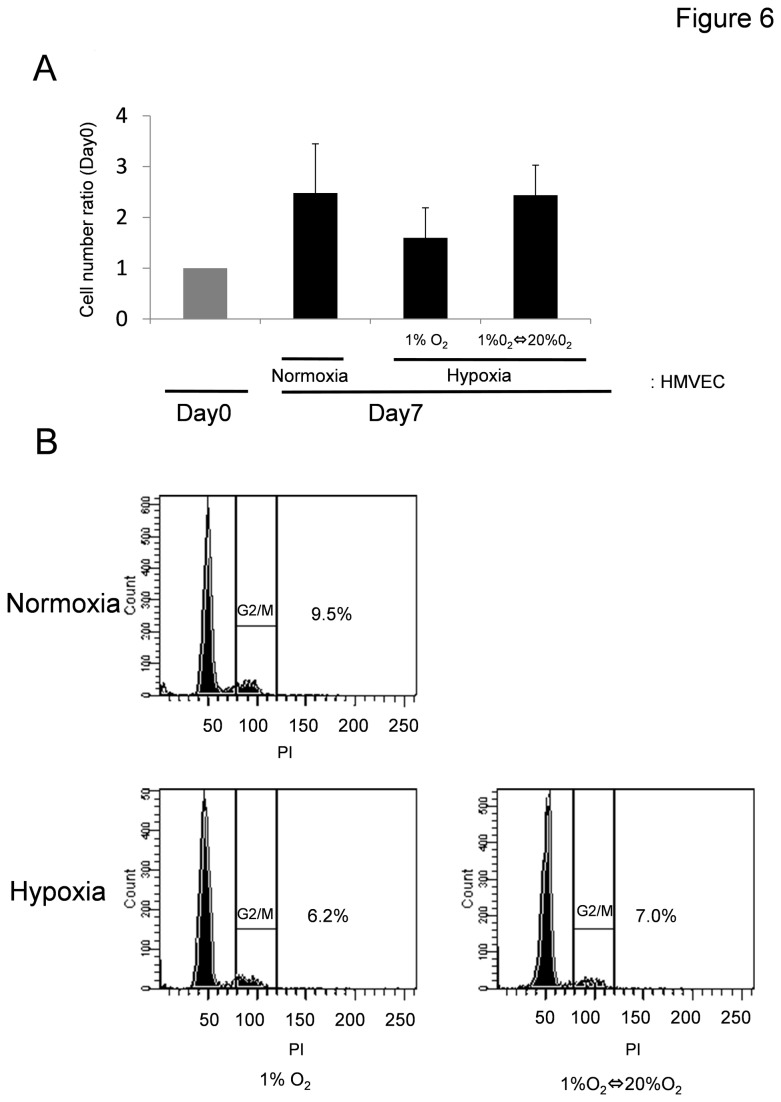
Association between cell cycle and EC aneuploidy in HMVECs exposed to hypoxia. (A) Cell number and the cell cycle in HMVECs exposed to hypoxia were analyzed by flow cytometry after staining fixed cells with propidium iodide. In each phase, cultured ECs proliferated for 7 days (normoxia, 2.5-fold; hypoxia (1% O_2_), 1.6-fold; hypoxia-reoxygenation, 2.4-fold versus Day 0 cell numbers). (B) We analyzed the distribution of ECs throughout the cell cycle by flow cytometry. There was no significant difference in cell-cycle distribution between conditions of normoxia and hypoxia. The experiment was repeated three times, and representative data is shown.

Genomic instability and cell-cycle checkpoints are associated [37]. In the cell-cycle arrest phase, some cells exhibit aneuploidy. G2-arrested cells frequently become aneuploid, presumably as a result of inappropriate reinitiation of DNA synthesis. To address the question of whether the increase in aneuploidy is due to cell cycle arrest, we analyzed cell proliferation and the distribution of ECs throughout the cell cycle by flow cytometry after staining fixed cells with propidium iodide. In each phase, cultured ECs proliferated for 7 days (normoxia, 2.5-fold; hypoxia, 1.6-fold; hypoxia-reoxygenation, 2.4-fold versus day 0 cell numbers; Figure 6A). Culture of ECs under conditions of hypoxia and hypoxia-reoxygenation did not change the relative distribution of living cells in Go/G1 and G2/M subsets, (Figure 6B). These results suggest that hypoxic stress did not affect the cell cycle of ECs and that hypoxia-induced aneuploidy was not due to G2 arrest. In fact, aneuploid mTECs proliferate well as we have already observed [16]. Furthremore, Ki67 staining demonstrated that aneuploid TECs *in vitro* (Figure S2) and *in vivo* (Figure S3) were proliferative. 

### Elevation of ROS induced by hypoxia and VEGF signaling cause aneuploidy in ECs

**Figure 7:**
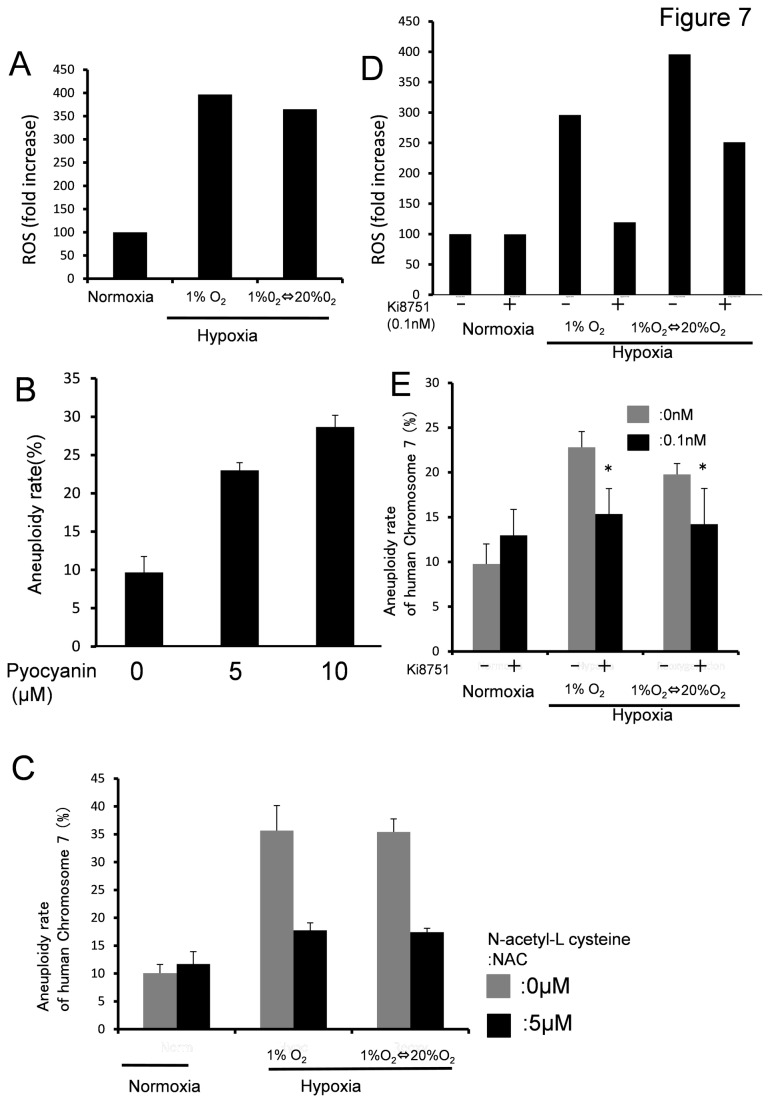
Hypoxia elevates ROS production and VEGF signaling increases aneuploidy in ECs exposed to hypoxia. (A) Cellular ROS in HMVECs were analyzed after exposure to normoxia, hypoxia (1% O2), and hypoxia-reoxygenation for 7 days. The experiment was repeated three times. Representative data is shown. (B) The ROS inducer pyocyanin increased EC aneuploidy in a concentration-dependent manner. (C) Inhibition of RO. (B) S by the ROS scavenger N-acetyl-cysteine (NAC) decreased hypoxia-induced aneuploidy in ECs. (D) Involvement of VEGF signaling in ROS production. Hypoxia-stimulated HMVECs reduced ROS production in the presence of the VEGFR inhibitor Ki8757. The experiment was repeated three times. Representative data is shown. (E) Aneuploidy induced by the hypoxic condition in HMVECs was significantly suppressed by Ki8751. *p < 0.05.

Genomic instability was related to elevated ROS levels [38]. To gain additional insight into aneuploidy in ECs exposed to conditions of hypoxia or hypoxia-reoxygenation, we assessed ROS levels by measuring dichlorofluorescein, the oxidation product of CM-H2DCFDA, by flow cytometry. The histogram of hypoxia-treated ECs or hypoxia-reoxygenation for 7 days showed a rightward shift compared with the FACS histogram of normoxia-treated ECs, indicating a more extensive oxidation of the dye inducing ROS (Figure 7A). 

The ROS inducer pyocyanin increased EC aneuploidy in a concentration-dependent manner (Figure 7B). In addition, inhibition of ROS by the ROS scavenger N-acetyl-cysteine (NAC) decreased hypoxia-induced aneuploidy in ECs. The results suggested that ROS were elevated in ECs by hypoxia. On the other hand, inhibition of ROS by NAC inhibited hypoxia-induced aneuploidy in ECs (Figure 7C). These results suggest that elevation of ROS by hypoxic stress in the tumor microenvironment may be one of the mechanisms by which TECs acquire chromosomal abnormalities. VEGF-induced Rac1-dependent NADPH oxidase activity, resulting in a transient increase in ROS levels, and NADPH oxidase-derived ROS are essential for the proliferation and migration of ECs [39,40]. ROS is directly involved in all these mechanisms, as H_2_O_2_ has been shown to induce proliferation and migration of ECs [39,40]. ROS also act as mediators of angiogenic growth factors such as VEGF [41].

High VEGF signaling increases chromosomal abnormalities through excess centrosome numbers in ECs in developing vessels [35]. ROS act as downstream messengers for VEGF and stimulate angiogenic signals to promote angiogenesis [42,43]. We further addressed the question of whether inhibition of VEGF signaling suppresses ROS production and aneuploidy of ECs in the hypoxic condition. A VEGFR inhibitor (Ki8751) significantly suppressed hypoxia-induced ROS production (Figure 7D) and decreased the aneuploidy rate in hypoxia-stimulated HMVECs (Figure 7E).

These results suggest that elevation of VEGF signaling by hypoxia was involved in the increase in ROS and that it caused aneuploidy in ECs.

## Discussion

Our previous comparison of TECs and NECs revealed many differences, including chromosome aberration and structural abnormalities of chromosomes, such as marker chromosomes of unknown origin, chromosome translocation, microchromosome pairing, and chromosome deletion in TECs [16,34,44].

In normal tissue, the vascular system functions through the maintenance of an equilibrium between angiogenesis and inhibitory factors. In tumor cells, however, excessive levels of proangiogenic factors (such as VEGF) are produced, and this results in a disorganized and extended immature vascular network. The permeability of new blood vessels is enhanced because of abnormal adhesion between the basement membrane and mural cells [45,46]. Subsequently, interstitial pressure rises in the tumor tissue and blood vessels bend, causing stagnation of blood flow, which is likely to cause hypoxia in the tissue. Moreover, unstable blood flow causes repeated temporary blockage and improvement of blood flow, which leads to sudden changes in oxygen level (reoxygenation) and, sometimes, genetic instability [36]. Under these conditions, some regions of blood vessels lack blood flow in the tumor microenvironment, suggesting that tumor ECs are also exposed to hypoxia. Because some features acquired by TECs in response to hypoxia are similar to the properties of tumor cells, we designed this study on the basis of the idea that the hypoxic environment in cancer may be the cause of TEC abnormality.

Our results demonstrated that human NECs (HMVECs) acquired aneuploidy in hypoxic conditions, suggesting that intratumor hypoxia is one of the mechanisms involved in the development of chromosomal aberrations in TECs.

Oxidative stress due to excess ROS production is associated with carcinogenesis, maintenance of the characteristics of cancer, and genetic instability of cancer cells. ROS production increases in cancer cells under hypoxic conditions [47], and increased ROS production is reported to cause genetic instability [38]. However, the association between ROS elevation in TECs and abnormalities of these cells has not been elucidated. In our study, hypoxic stress led to accumulation of ROS in human NECs and induced aneuploidy in them. These results suggest that hypoxia-induced ROS accumulation is involved in chromosomal abnormality in tumor vascular ECs. It is still questionable whether hypoxia is the only mechanism. For example, EC aneuploidy has not been reported in ischemic lesion. Because HMVECs were cultured in endothelial culture medium (EGM-2 MV) containing high concentration of growth factors in our current study, growth factors in microenvironment may be other mechanism for EC aneuploidy.

In addition to hypoxia, growth factors such as VEGF are involved in ROS production. Therefore, ROS production increases because of enhanced VEGF signaling in human NECs (HUVECs), and exposure of cancer cells to hypoxia due to ischemia produces angiogenic factors, including VEGF, with subsequent incorporation of surrounding normal blood vessels [41]. We previously reported that the expression of angiogenic factors such as VEGF is also upregulated in TECs, and this contributes to their high viability through an autocrine mechanism. The findings of the present study suggest that activation of VEGF signaling in TECs under hypoxia may cause chromosomal aberrations in TECs through ROS accumulation.

Resistance to antiangiogenic drug therapy has recently been reported [48]. A phenotypic change in tumor cells has been considered to be the most likely mechanism for this. However, we recently reported that ECs could acquire drug resistance by upregulation of MultiDrug Resistance gene (MDR1) resulting from exposure to a high level of VEGF [33].

The results of the present study indicate that NECs may also acquire chromosomal abnormalities by ROS accumulation due to hypoxia followed by activation of VEGF signaling. Therefore, acquisition of chromosomal instability and drug resistance in TECs may be caused by a hypoxic microenvironment in the tumor.

To overcome drug resistance in anti-angiogenic therapy, it is therefore important to normalize hypoxia in tumor tissue. There is also current interest in a newly proposed concept in anti-angiogenic therapy: normalization of tumor vasculature. Using anti-VEGF drugs, immature blood vessels in the tumor are normalized and tumor hypoxia is overcome. After tumor tissue becomes normoxic, radiation therapy is more effective and anticancer drugs are delivered more easily because of the normalized and organized vasculature. This is also an important strategy in the administration of anti-angiogenic therapy. We have demonstrated that hypoxia causes cytogenetic abnormalities in ECs through ROS accumulation. Therefore, restoration of the hypoxic condition in tumors may be a potential strategy for overcoming resistance in anti-angiogenic therapy targeting TECs as well as tumor cells. Actually, we observed TEC isolated from low metastatic tumor, in which blood vessels show mature structure, and which was not hypoxic, were diploid [15]. Since continuous stimulation of excess amount of VEGF and hypoxia may affect chromosomal abnormality, vascular normalization could prevent for chromosomal abnormality in TECs. NAC and epigallocatechin-3-gallate (EGCG) are antioxidant compounds and ROS scavengers; therefore, they may be helpful pharmacological tools to inhibit TEC abnormalities by scavenging endogenous ROS in these cells. Inhibition of TEC abnormalities by normalizing hypoxia through the inhibition of ROS accumulation using anti-VEGF therapy, HIF inhibitors, and/or antioxidants may reverse drug resistance to anti-angiogenesis therapy.

## Supporting Information

Figure S1 **Primers used for RT-PCR analysis.**
(TIF)

Figure S2 **Expression of Ki67 in mTECs.** (A) Cultured TECs were stained with anti-Ki67 (green) Nuclei are stained with DAPI (blue). TECs cultured in EGM-2MV were Ki67-positive. (B) Cultured TECs were stained with anti-Ki67, and FISH was performed using a Cy3-mouse chromosome-17 locus-specific BAC probe. Most of TECs have aneuploidy. Scale bar, 25 μm.
(TIF)

Figure S3 **Expression of Ki67 in tumor tissue.** (A) Tumor tissue was stained with anti-Ki67 (green) and anti-CD31 (red). Aneuploidy was observed in ECs of Ki67-positive tumor blood vessels.
(TIF)
