# Supplementary figures and images for: Hypoxia-Induced Reactive Oxygen Species Cause Chromosomal Abnormalities in Endothelial Cells in the Tumor Microenvironment
Source: PLoS One. 2013 Nov 15;8(11):e80349. doi: 10.1371/journal.pone.0080349 (PMC3829944; doi:10.1371/journal.pone.0080349)

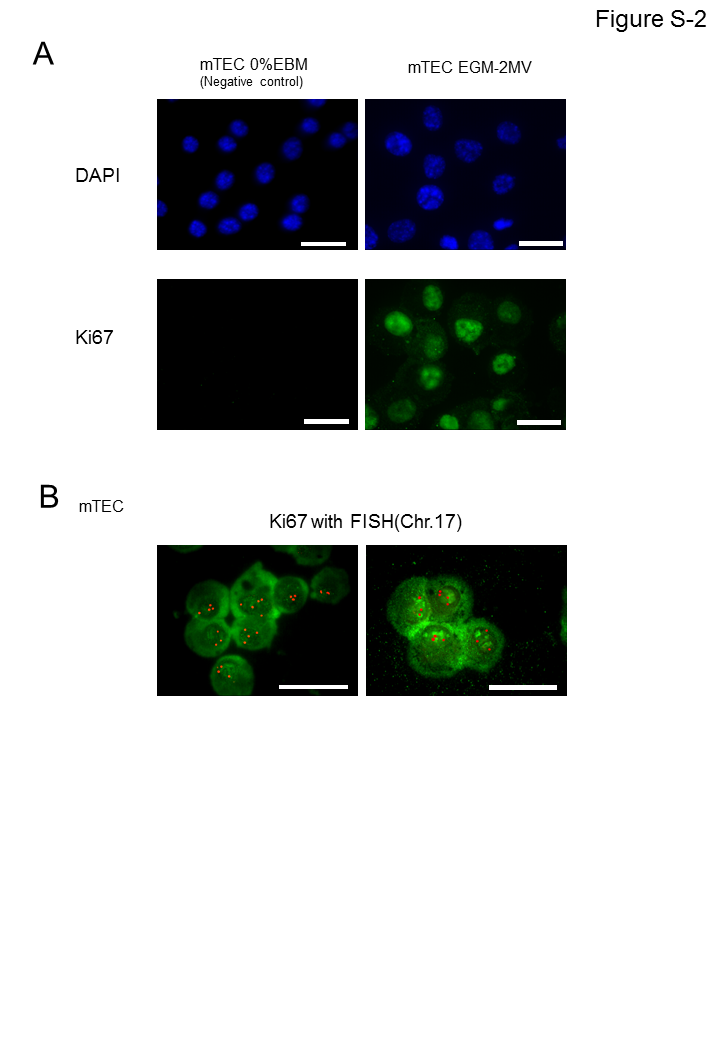

Supplement: Figure S2 — Expression of Ki67 in mTECs. (A) Cultured TECs were stained with anti-Ki67 (green) Nuclei are stained with DAPI (blue). TECs cultured in EGM-2MV were Ki67-positive. (B) Cultured TECs were stained with anti-Ki67, and FISH was performed using a Cy3-mouse chromosome-17 locus-specific BAC probe. Most of TECs have aneuploidy. Scale bar, 25 μm. (TIF) [file pone.0080349.s002.tif]

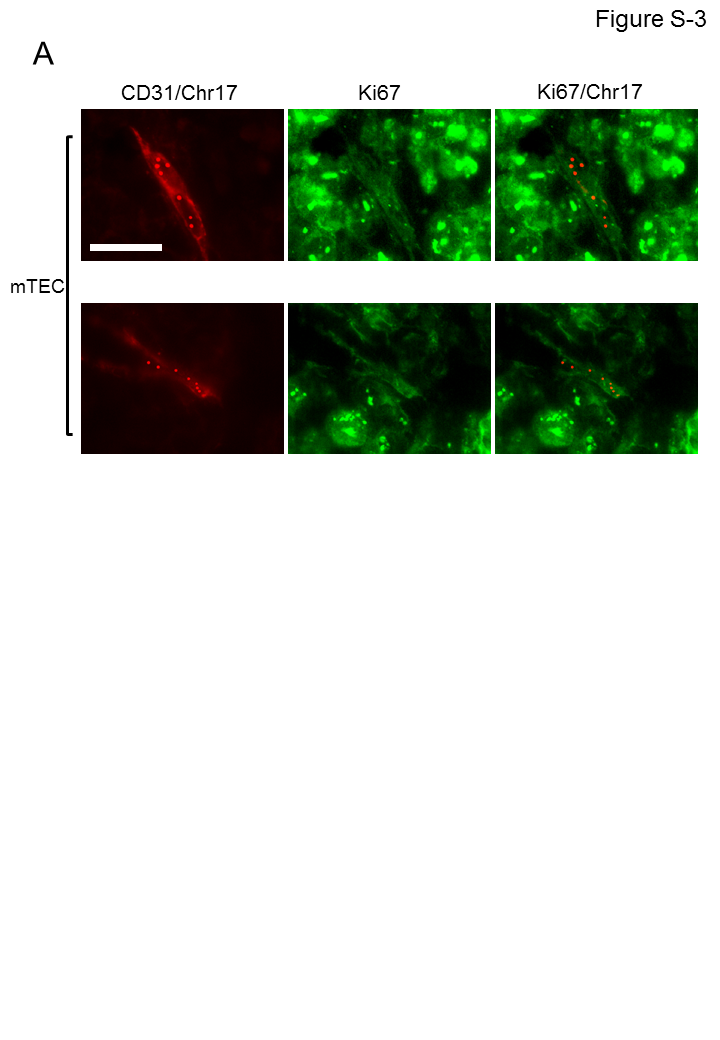

Supplement: Figure S3 — Expression of Ki67 in tumor tissue. (A) Tumor tissue was stained with anti-Ki67 (green) and anti-CD31 (red). Aneuploidy was observed in ECs of Ki67-positive tumor blood vessels. (TIF) [file pone.0080349.s003.tif]
